# A Highly Efficient Aromatic Amine Ligand/Copper(I) Chloride Catalyst System for the Synthesis of Poly(2,6-dimethyl-1,4-phenylene ether)

**DOI:** 10.3390/polym10040350

**Published:** 2018-03-22

**Authors:** Kisoo Kim, Min Jae Shin, Yong Tae Kim, Joong-In Kim, Young Jun Kim

**Affiliations:** 1 School of Chemical Engineering, Sungkyunkwan University, 2066 Seobu-ro, Jangan-gu, Suwon-si, Gyeonggi-do 16419, Korea; kks0918@hyosung.com (K.K.); shinminjae@lgcare.com (M.J.S.); 2 Hyosung R&D Business Labs, 74 Simin-daero, Dongan-gu, Anyang-si 14080, Gyunggi-do, Korea; 3 LG Household & Health Care R&D Center, LG Science Park, Magok, Kangseo-go, Seoul 07795, Korea; 4 SAMSUNG SDI, SDI-dong, Maetan-dong 130, Samsung-ro, Yeongtong-gu, Suwon-si, Gyeonggi-do 16678, Korea; yt74.kim@samsung.com; 5 Hyosung R&D Business Labs, 74 Simin-daero, Dongan-gu, Anyang-si, Gyunggi-do 14080, Korea; joonginkim@hyosung.com

**Keywords:** rate of polymerization, aromatic amine ligand, highly effective catalyst, copper(I) chloride, poly(2,6-dimethyl-1,4-phenylene ether), 3,3′,5,5′-tetramethyl-4,4′diphenoquinone

## Abstract

Highly active catalyst systems for polymerizing 2,6-dimethylphenol were studied by using aromatic amine ligands and copper(I) chloride. The aromatic amine ligands employed were pyridine, 1-methylimidazole, 2-aminopyridine, 3-aminopyridine, and 4-aminopyridine. A mixture of chloroform and methanol (9:1, *v*/*v*) was used as a polymerization solvent. All experiments were performed with oxygen uptake measurement apparatus, while the reaction rate for each aromatic amine ligand-Cu catalyst system and the amount of by-product, 3,3′,5,5′-Tetramethyl-4,4′diphenoquinone (DPQ), were measured to determine the efficiency of the catalyst systems. The 4-aminopyridine/Cu (I) catalyst system was found to be extremely efficient in poly(2,6-dimethyl-1,4-phenylene ether) (PPE) synthesis; it had the fastest reaction rate of 6.98 × 10^−4^ mol/L·s and the lowest DPQ production. The relatively high basicity of 4-aminopyridne and the less steric hindrance arising from a coordination of Cu and 4-aminopyridine in this catalyst are responsible for the fast polymerization rate. When 2-aminoprydine (an isomer of 4-aminopyridine) was used as a ligand, however, no polymerization occurred probably due to steric hindrance.

## 1. Introduction

Poly(2,6-dimethyl-1,4-phenylene ether) (PPE) is one of the most important engineering plastics, with a unique combination of mechanical properties, low moisture absorption, an excellent electrical insulation property, dimension stability, and inherent flame resistance [1]. Even though PPE has the melting temperature of 236 °C, it is generally classified as an amorphous polymer with the glass transition temperature of 205 °C since its degree of crystallinity is as small as 3% [2]. It is often processed with polystyrene due to its high softening point and high melt viscosity [3,4,5] and is widely used in many industrial applications, such as computer housings, television housings, keyboard frames, and interface boxes [1,6,7].

**Figure 1:**
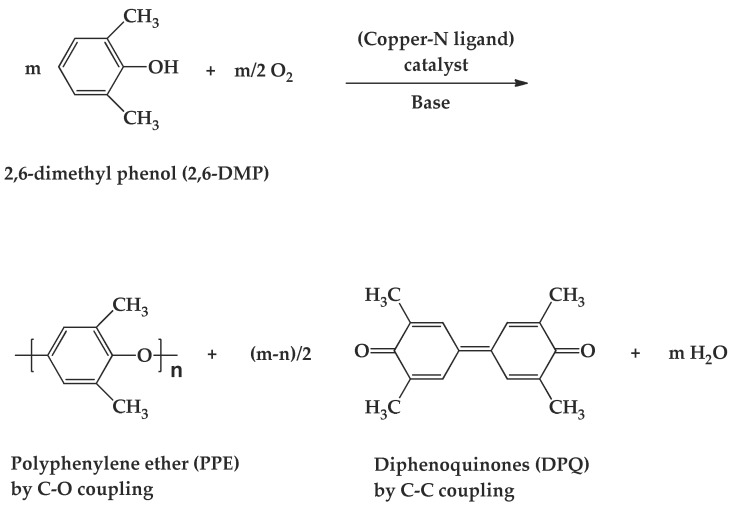
Synthesis of poly(2,6-dimethyl-1,4-phenylene ether) (PPE) by the copper-catalyzed oxidative coupling of dimethylphenol (2,6-DMP).

Hay and co-workers have reported a synthesis method for PPE in which the monomer, 2,6-dimethyl phenol, in the CuCl/pyridine catalyst system yields high molecular weight PPE with the flow of O_2_ [8]. As shown schematically in Figure 1, the major product, PPE, is formed via C–O coupling and the minor product, 3,3′,5,5′-tetramethyl-4,4′diphenoquinone (DPQ), is formed via C–C coupling at a yield less than 5% under proper polymerization conditions with the formation of the condensation product water [9].

Although the synthesis mechanism has been studied by a significant number of researchers due to its excellent physical properties and being feasible, the mechanism actually has remained unclear. Proposed mechanisms are the radical mechanism, which involves free phenoxy radicals and coordinated phenoxy radicals, and the ionic mechanism, which involves ions such as phenoxonium cation [10,11]. Chloroform and toluene are generally used as polymerization solvents, and recently the usage of water as a solvent is being studied as one of the polymerization methods in respect of considering the environment [12,13,14,15]. Limiting the amount of DPQ production is critical, since the remaining DPQ in polymers plays a detrimental role that accelerates the decomposition of PPE while processing it at high temperature. For the ligands, aliphatic amines and imidazole and its derivatives are widely used due to their capability to bind with metal catalysts and the frequent occurrence of C–O coupling during PPE synthesis [16,17]. Studies have indicated that the remaining ligands in the polymers may lower the thermal stability of the PPE and change the color, especially when aliphatic amine ligands are used. This was verified by ^13^C-NMR spectroscopic analysis, and the most persuasive suggestion is that the quinone methide intermediate can be formed during PPE synthesis and it quickly reacts with an amine to remain in the products [18]. The incorporated amines could then deteriorate the thermal stability and cause a color change during processing.

Considering the previously mentioned aspects, different types of aromatic ligands were employed in this study instead of aliphatic amine, which can be contained in a polymer, to aim for developing a highly active aromatic amine/Cu catalyst system with a fast reaction rate and that is low in DPQ production.

## 2. Experimental

### 2.1. Materials

Highly purified (>99.9%) 2,6-dimethylphenol (2,6-DMP) was provided by Aekyung Petrochemical (Daejeon, Korea) and copper(I) chloride (CuCl) (99.995%), chloroform (99.8%), methanol (99.8%), 1-methylimidazole (99%) (L_1_), 3-aminopyridine (99%) (L_3_), pyridine (99%), and citric acid (99.5%) were purchased from Sigma Aldrich Korea (Seoul, Korea). 2-Aminopyridine (99% pure) (L_2_) was purchased from Alfa Aesar Korea (Seoul, Korea) and 4-aminopyridine (98% pure) (L_4_) was purchased from Kanto Chemical Korea (Seoul, Korea). 3,3′,5,5′-tetramethyl-4,4′diphenoquinone (DPQ) (98%) was purchased from Sigma Aldrich and was recrystallized in a needle-shape with a mixed solvent of dichloromethane/n-hexane, 1:1 (*v*/*v*), and dried overnight in a vacuum oven at 80 °C.

### 2.2. Oxidative Coupling Polymerization of PPE and Oxygen Uptake Measurements

All 2,6-DMP polymerization was implemented with the oxygen uptake measurement apparatus and an attached flask which was manufactured by our own laboratory [15]. Put 2,6-DMP 1 g (0.819 mmole), chloroform 9 mL, and a magnetic stir bar into a 500 mL three-neck round-bottomed flask. Connect a dropping funnel to the three-neck round-bottomed flask, and put the reagents-containing flask into the 25 °C thermostat connecting to the oxygen uptake measurement equipment. Stir at 700 rpm with oxygen flow at a rate of 330 cc/min in order to dissolve the monomer. After 5 min, put 1 mL of the ligand-dissolved methanol into a dropping funnel and set the oxygen measurement equipment to a closed-system. As the ligands-dissolved methanol is transferring from the dropping funnel to the three-neck round bottomed flask, start the 2,6-DMP polymerization and let the reaction take place for two hours. Record the amount of oxygen uptake from the beginning to the end of the polymerization in 30-s intervals. After an hour of polymerization, the rate of oxygen uptake was very slow, but the polymerization was conducted for 2 h in a conservative manner. After 2 h of polymerization, precipitate the liquid products into the methanol with 1 g of citric acid dissolved in it, then retrieve. Dry overnight at 100 °C in a vacuum oven and collect the PPE powder. In order to investigate the effect of ligand and CuCl molar concentration on the polymerization reaction, the molar ratios of DMP, ligand, and CuCl have been varied as shown in Table 1.

### 2.3. Measurement of DPQ Content

**Figure 2:**
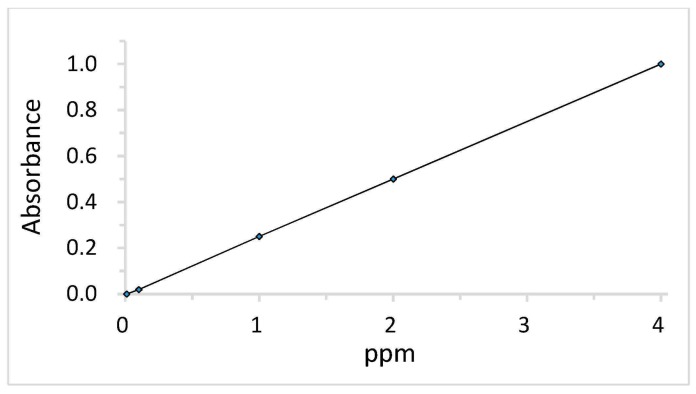
A plot of 3,3′,5,5′-Tetramethyl-4,4′diphenoquinone (DPQ) concentration (ppm) versus absorbance for determining wt % of DPQ.

Since DPQ, the by-product of PPE synthesis, absorbs light in a wavelength of ca. 421 nm, the amount of product was determined by measuring absorbance at 421 nm with ultraviolet (UV) spectroscopy [19,20]. The calibration curve was obtained from measuring UV absorbance at λ_max_ = 421 nm of a DPQ at the concentration of 0.01, 0.1, 1, 2, and 4 ppm in chloroform (Figure 2). After the reaction was completed, 0.1 mL of polymerization mixture in a liquid state was collected and weighed. Then, the polymerization mixture was diluted in 100 mL of chloroform for UV spectroscopic measurements and the absorbance was measured in order to analyze the DPQ contents in the samples based on the calibration curve.

### 2.4. Calculation of the Number of Moles of Oxygen Being Reacted during Polymerization Reaction

Since the monomer, 2,6-DMP, and oxygen are reacting in a ratio of 2 to 1, the number of moles of 2,6-DMP reacted can be calculated by measuring the oxygen uptake during the reaction, and then the reaction rate can also be determined by the moles of consumed 2,6-DMP. The density of oxygen gas at 25 °C was calculated from the following Equation (1) [21], and the number of moles was obtained from the density.

(1) $$rO_{2}^{t}=\left(rO_{2}^{0}p\right)/\left\{760\times \left(1+\alpha t\right)\right\}$$

where r$O_{2}^{t}$ = dioxygen density (g/L) at t °C, r$O_{2}^{0}$ = dioxygen density (g/L) at 0 °C (= 1.429 g/L), α = thermal expansion coefficient (=0.00367 K^−1^), and *p* = atmospheric pressure (mmHg).

### 2.5. Determination of Molecular Weight of the Polymers and Dilute Solution Viscosity

The molecular weight of the polymers was measured by K-802, K-803, and K-804 (Shodex) columns attached to a gel permeation chromatography (GPC) with chloroform as a solvent at 30 °C in a flow rate of 0.5 mL/min. The molecular weight and polydispersity index (PDI), were then analyzed by using polystyrene standards (Waters). To determine the intrinsic viscosity ([η]), the flow times of dilute solutions were measured using a Cannon-Ubbelohde viscometer (size 0 B) (State College, PA, USA) in CHCl_3_ at 30 °C.

## 3. Results and Discussion

### 3.1. Effects of Chemical Structure of Ligand on the Rate of Polymerization

**Figure 3:**
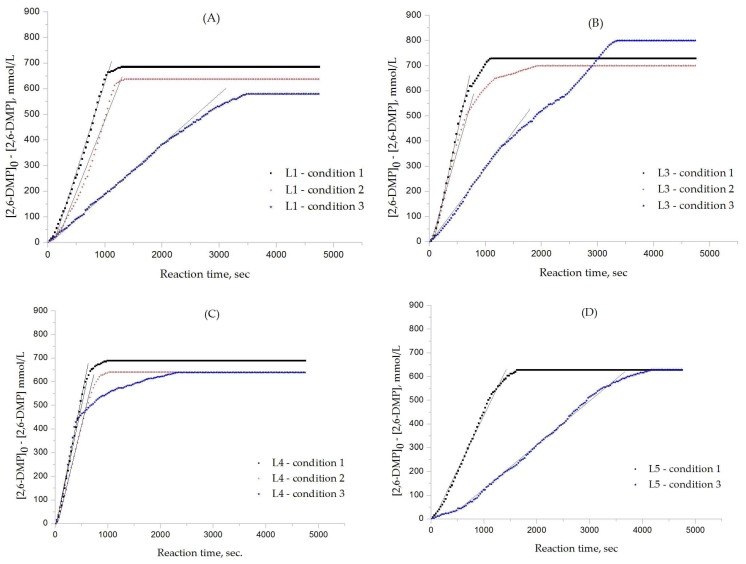
Plots of decrease in molar concentration of 2,6-DMP (mmol/L) versus reaction time (s): (**A**) for L_1_; (**B**) for L_3_; (**C**) for L_4_; (**D**) for L_5_.

Figure 3 shows plots of decrease in molar concentration of 2,6-DMP (mmol/L) versus reaction time (s) for L_1_, L_3_ and L_4_, L_5_, respectively, where [2,6-DMP]_0_ is the initial molar concentration (=818.6 mmol L^−1^) and [2,6-DMP] is the molar concentration given time. The rates of decrease in 2,6-DMP concentration were obtained from the slopes of the plots where linearity holds in a wide range. The rate of decrease in 2,6-DMP concentration also includes the rate of DPQ formation, which would be approximated to the rate of polymerization if the amount of DPQ produced is small.

The effects of the chemical structure of each ligand on the rate of polymerization are presented in Table 2. These observations could be interpreted in view of Cu concentration, basicity, and steric effect of the ligands. For all the ligands used, the reaction rates tend to increase in the order of Condition 1, 2, and 3. This means that the PPE polymerization rate is more affected by the amount of Cu catalyst under the experimental conditions performed than that of the ligands. As the ligand basicity increases, the polymerization rate tends to increase, except for L_1_ and L_2_, which have not polymerized. The ligands have not only affected the formation of the coordination complex with the Cu ion, but also the solvent basicity, which significantly changes the polymerization rate probably by affecting the oxidation potential of the phenol. A ligand with high basicity is strongly combined to the Cu metal pre-catalysts and it cannot be easily released. Therefore, amine ligands should have appropriate basicity in order to have high catalytic activity. The basicity of ligands is shown in Table 3.

The amino pyridine isomers L_2_, L_3_, and L_4_ have conspicuously different effects on the polymerization rate. It is also observed that m-aminopyridine (L_3_) or p-aminopyridine (L_4_) is a more effective ligand than N-methylimidazole (L_1_), which is perhaps the most widely employed aromatic ligand [15,16,17]. When L_4_ is used, the fastest polymerization rate of 6.98 × 10^−4^ mol/L·s is observed. Polymerization did not occur for L_2_ although its basicity is similar to that of L_1_ and a faster reaction rate was observed for L_4_ than for L_3_.

**Figure 4:**
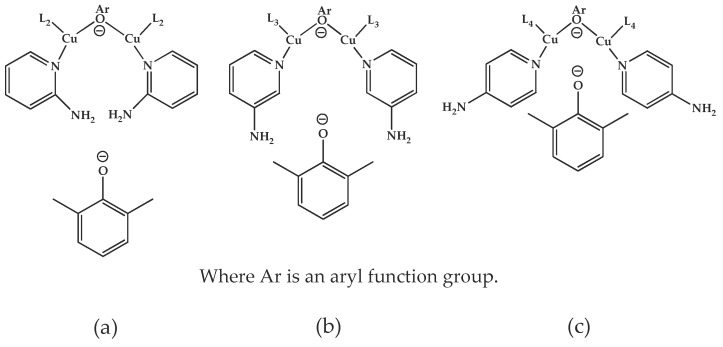
Steric environments of coordination compounds derived from L_2_, L_3_, or L_4_: (**a**) for L_2_, approach of the phenolate anion to the Cu ion is impossible due to strong steric hindrance (no polymerization); (**b**) for L_3_, approach of phenolate anion is possible due to reasonably relieved steric hindrance (polymerization occurs); (**c**) for L_4_, easy access of phenolate anion free from steric hindrance (fast polymerization occurs).

The effects of isomeric L_2_, L_3_, and L_4_ ligands on the rate of polymerization are very different, which could be speculated on in view of steric effects [19]. Figure 4 shows the accessibility of a phenolate anion when L_2_, L_3_, and L_4_, which are ortho-, meta-, and para-positioned, respectively, are coordinated to a Cu ion. The steric hindrance which occurs during the formation of the Cu–amine ligand complex is a possible reason for the result. A possible active catalytic species in PPE synthesis is a coordination compound formed from two copper ions, amine ligands, and an aryloxide anion. When a Cu–amine ligand complex is formed, the steric hindrance from the ligands would make it difficult for the phenolate anion to approach the Cu ion, and the reactivity would decrease as a result. In the case of L_2_, when the phenolate anion is about to coordinate with the Cu catalyst, it is subjected to steric hindrance from the amino group at the ortho position of L_2_. Therefore, it appears that in all of the experiments using L_2_ polymerization does not occur. Since L_4_ is the most basic ligand and free from steric hindrance in the formation of the coordination compound, it shows the highest reaction rate. L_3_ has a weaker basicity than L_1_, but leads to a larger polymerization rate. This observation could not be rationalized by steric hindrance since L_3_ would experience similar or less steric hindrance than L_1_ during a complex formation process. In fact, the rate of polymerization is greatly influenced by the type of solvent in addition to basicity and steric hindrance [25]. These two ligands differ in polarity: L_1_ is a polar aprotic compound and L_3_ is a polar protic compound. This difference seems to have affected the polymerization rate.

### 3.2. Effects of Ligand and Polymerization Conditions on DPQ Formation and Molecular Weight of Polymer

In Table 4, the amount of DPQ produced for each experiment is shown. The result of L_2_ has been omitted for its not being polymerized. When L_4_ was used, the lowest DPQ formation was obtained at 2.2%, and similar results were obtained under different experimental conditions. It is reported that the formation of DPQ is affected by various parameters, such as the polymerization temperature, types of solvent, polarity of reaction medium, and basicity of ligands, and its formation significantly increases as both solvent acidity and polymerization temperature increase [8,16,26,27,28]. DPQ production is the lowest with L_4_, which has the fastest reaction rate. This observation might be due to a low monomer concentration at the initial stage of polymerization as a result of the fast polymerization rate. As reported elsewhere [10], DPQ is largely produced at the beginning of the reaction when the concentration of the monomers is the highest since DPQ is formed by the C-C coupling of two 2,6-DMP monomers. It is also observed that the amount of ligand affects the production of DPQ more than the amount of Cu catalyst within the experimental conditions. The experimental result of Table 4 shows that the amount of DPQ formed is the biggest under Condition 2 for every ligand. It is presumed that the amine ligand changed the basicity of the solution and affected the production of DPQ.

The yield, viscosity, and molecular weight of produced PPE in each experiment are presented in Table 5. As previously mentioned, L_2_ and L_5_ have not polymerized in Condition 2, while PPE in high molecular weight could be obtained in all the other experiments, and the highest molecular weight was acquired with L_3_. In particular, L_3_, which has lower basicity than L_4_, resulted in high molecular weight polymers despite slow polymerization, which can be speculated on as follows. In fact, the 2,6-DMP polymerization is known to behave as a step-growth polymerization [29]. One of the important characteristics of the step-growth polymerization is that a high molecular weight polymer is achieved at the very end of polymerization. As the polymerization proceeds, the concentration of OH^−^ ions formed from the equilibrium reaction of H_2_O and the ligand increases, and the greater the basicity of the ligand, the greater the concentration of OH^−^ ions. At the end of the polymerization, the concentration of OH^−^ can be significantly high, especially for the ligands having strong basicity since the concentration of fairly acidic phenolic OH groups (pKa ~ 10.2) is low. The OH^−^ ions also play a role in lowering the oxidation potential of 2,6-DMP, which makes the oxidative polymerization easier. However, when the OH^−^ ion concentration exceeds a certain level, the concentration of the active catalytic species decreases and the rate of polymerization decreases [30]. That is, in the case of L_4_, which has higher basicity than L_3_, the polymerization rates were perhaps significantly lowered at the end of the polymerization by ‘catalyst poisoning’. Thus, L_4_ resulted in lower molecular weight polymers than L_3_. L_4_ under Condition 2 (the condition with half the concentration) resulted in higher molecular weights than under Conditions 1 and 3. L_5_ resulted in higher molecular weights under Conditions 1 and 2 than L_4_. These observations can be explained for the same reason explained above. L_3_ resulted in higher molecular weights than the other ligands probably because it is an effective ligand leading to a fast reaction rate and the basicity was not too large, so catalyst poisoning did not occur until the end of the reaction.

In Table 5, the PDI values appear in a wide range of 1.6–3.5. PDI values in a wide range have also been reported elsewhere [13]. It is not easy to explain this observation, since the polymerization mechanism is unclear at this point. However, it could be rationalized by considering the fact that the polymerization mechanism involves both a radical pathway and an ionic pathway [31], which may lead to molecular weight distributions different from those of conventional step-growth polymerization.

## 4. Conclusions

In this study, PPE was synthesized under three conditions with various aromatic amine ligands, including 1-methylimidazole, which is widely used for PPE synthesis. As a result, the 4-aminopyridine/Cu (I) catalyst system was the most efficient catalytic system for PPE synthesis as it had the fastest reaction rate and the lowest DPQ production under the experimental conditions. This is due to the adequate basicity of 4-aminopyridine and less steric hindrance as a phenolate anion approaches a Cu ion to form an active catalytic complex. With adequate basicity and less steric hindrance depending on the ligand structure, the reaction rate increases. Since the formation of DPQ usually occurs during an early stage of the reaction, it appears to have a lower amount of DPQ production as the reaction rate increases. The observed molecular weight dependence on polymerization conditions suggests that it is necessary not only to use a ligand leading to a fast reaction rate but also to control the basicity of the polymerization medium to prevent catalyst poisoning and to synthesize high molecular weight polymers.

## Figures and Tables

**Table 1:** Mole ratios of 2,6-DMP, ligand, and CuCl employed in polymerization.

Condition	2,6-DMP (g)	Molar ratios		
		[2,6-DMP]:[Ligand]:[CuCl]		
1	1	200	70	2
2		200	35	2
3		200	70	1

**Table 2:** Effects of chemical structure of ligands on the rate of polymerization.

		Rate of Polymerization (10^-4^ mol/L·s)				
	Ligand	L_1_	L_2_	L_3_	L_4_	L_5_
Condition		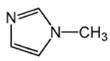	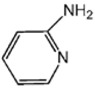	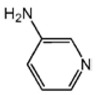	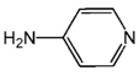	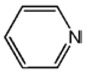
1		5.40	X *	6.53	6.98	3.89
2		4.73	X	3.57	6.58	X
3		1.68	X	2.38	2.77	1.51

X *: No polymerization.

**Table 3:** pKa values of amine ligands.

	Ligands	pKa
L_1_	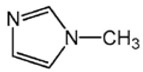	6.95 [22]
L_2_	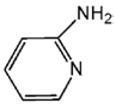	6.86 [23]
L_3_	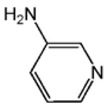	5.80 [23]
L_4_	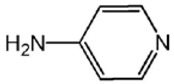	8.96 [23]
L_5_	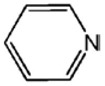	5.14 [24]

**Table 4:** Weight % of DPQ in PPE.

Condition	L_1_	L_3_	L_4_	L_5_
1	4.3	4.2	2.2	4.4
2	10.4	7.7	2.8	15.7
3	3.3	3.9	2.5	5.7

**Table 5:** Effects of ligand and polymerization condition on molecular weight of PPE.

Ligand	Condition	Yield (%)	Intrinsic Viscosity (dL/g)	*M_n_*	*M*_w_	PDI
L_1_	1	74.6	0.63	12,400	36,800	2.97
	2	75.6	0.60	8700	22,800	2.62
	3	69.5	0.20	8100	13,500	1.67
L_2_	1	X *	X	X	X	X
	2	X	X	X	X	X
	3	X	X	X	X	X
L_3_	1	82.6	0.84	15,400	44,000	2.86
	2	74.6	0.39	14,200	33,700	2.37
	3	96.7	1.02	16,100	44,500	2.76
L_4_	1	78.6	0.45	9,060	31,500	3.48
	2	72.6	0.36	13,600	32,700	2.40
	3	72.6	0.29	12,700	29,400	2.31
L_5_	1	61.5	0.40	12,400	29,400	2.37
	2	X	X	X	X	X
	3	64.5	0.59	14,900	38,900	2.61

X *: No Polymerization. PDI: polydispersity index.
